# Brown Tumor as a Reversible Cause of Osteolytic Lesions in Post-trauma Knee Pain: Role of Timely and Serial Parathyroid Hormone Testing

**DOI:** 10.7759/cureus.88199

**Published:** 2025-07-17

**Authors:** Monica Potru, Jitesh Rawat, Kanika Khandelwal

**Affiliations:** 1 Department of Radiology, Dr. Rajendra Gode Medical College, Amravati, IND; 2 Internal Medicine, Mayo Clinic Health System, Austin, USA

**Keywords:** brown tumor, hyperparathyroidism, knee pain, lytic lesions, metastasis differential, parathyroid adenoma, parathyroid hormone (pth)

## Abstract

Osteitis fibrosa is a metabolic skeletal disorder characterized by increased osteoblast and osteoclast activity, leading to abnormal bone matrix formation secondary to elevated parathyroid hormone (PTH) levels. Brown tumor is a rare and late manifestation of this condition that presents with focal well-defined osteolytic lesions, similar to metastatic disease. We present a case of a 52-year-old male with right knee pain following trauma. X-ray imaging demonstrated lytic lesions and a pathological fracture, ultimately leading to a diagnosis of a brown tumor. This case emphasizes the need to include brown tumors in the differential diagnosis of lytic bone lesions in middle-aged patients and highlights the role of serial PTH testing in identifying this uncommon condition.

## Introduction

A middle-aged male patient presented with knee pain following a ground-level fall. Imaging revealed osteolytic bone lesions, and he had normal parathyroid hormone (PTH) levels in the initial laboratory findings, which misdirected the diagnosis to metastasis. A repeat PTH level is a high rising suspicion for brown tumor in the right time before any possible aggressive management for malignancy. A brown tumor is a misnomer, and it is a rare manifestation of hyperparathyroidism, arising from increased bone resorption due to elevated PTH levels. Brown tumors occur in only 2-3% of patients with hyperparathyroidism and predominantly affect the mandible, clavicle, ribs, pelvis, and long bones [1]. These osteolytic lesions can be mistaken for metastatic lesions, highlighting the importance of accurate diagnosis to prevent inappropriate management. Diagnosis typically involves a combination of clinical, radiological, and laboratory evaluations, and elevated PTH is associated with brown tumors. However, high parathyroid harmone levels are not specific to brown tumors and can also be associated with other bone conditions like sub-periosteal resorption, osteitis fibrosa cystica, and osteoporosis. The diagnosis of a brown tumor was challenging because of the normal initial PTH level. This case highlights the importance of repeat PTH level testing, especially in middle-aged patients who have a relatively high chances of brown tumors. The serial PTH should also be considered in cases of osteolytic lesions suspected to be metastasis without a proven source of primary site of malignancy.

## Case presentation

A 52-year-old male presented to the emergency department with severe right knee pain following a fall. His medical history included right-sided sciatica, but no other significant comorbidities were noted. Initial laboratory results showed normal values for complete blood count, metabolic profile, blood urea nitrogen (BUN), creatinine, total protein, albumin, and PTH levels.

**Figure 1:**
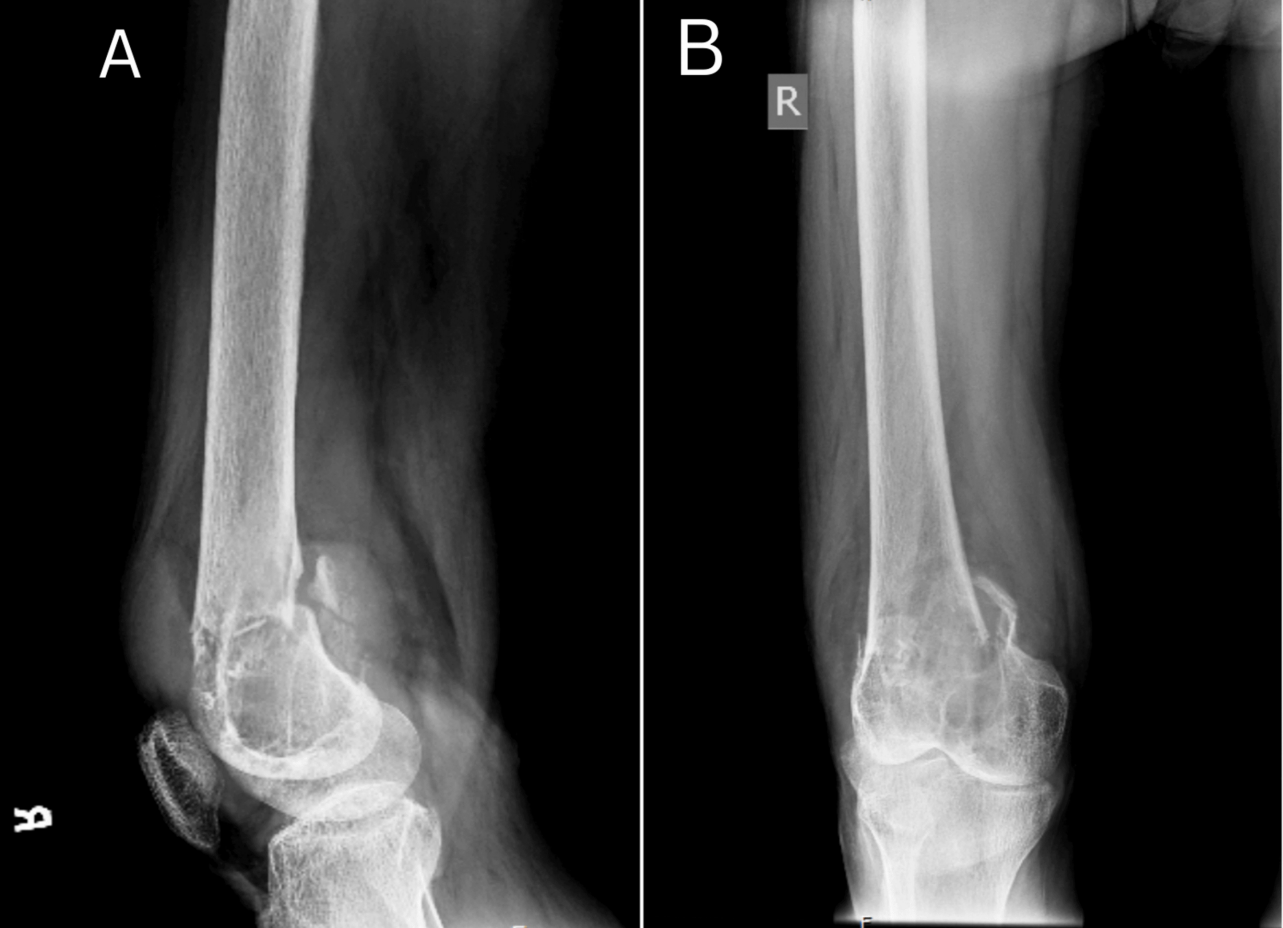
X-ray of the right femur (A) lateral and (B) AP showed a well-defined eccentric lytic lesion seen in the metadiaphyseal region of the distal end of the femur with a fracture of the bony fragment on the medial margin of the femur, which is displaced posteromedially. The lytic lesion involved a fracture of the lateral margin of the femur, leading to overlapping of the bone. No involvement of join margin is seen.

**Figure 2:**
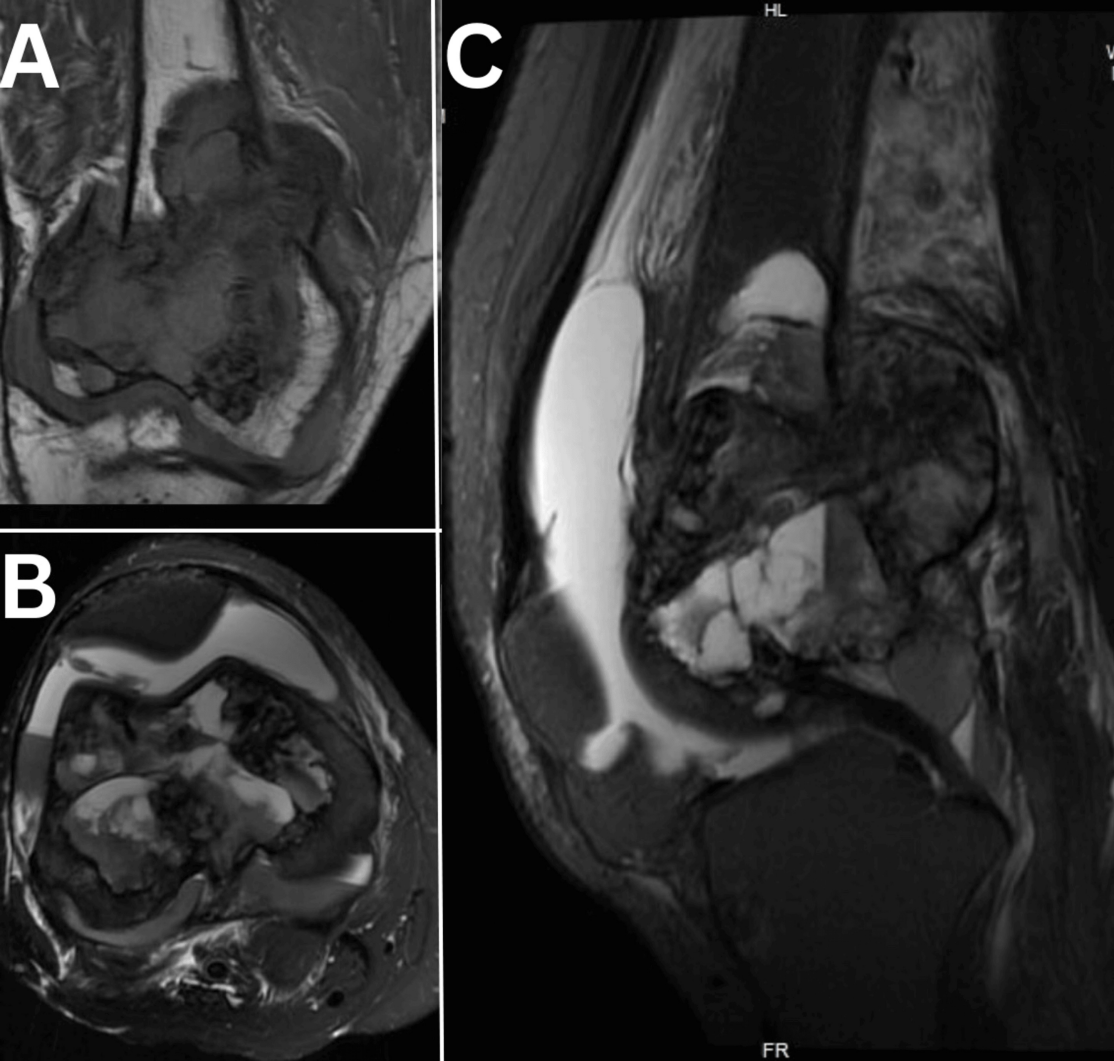
MRI of the right knee demonstrating (A) the coronal T1-weighted image (T1WI), (B) axial fat-suppressed (FS) T2-weighted image (T2WI), and (C) sagittal FS T2WI. These images reveal a well-defined, multilobulated, expansile lesion in the subarticular region of the distal femoral condyle. The lesion appears iso- to hypointense on T1WI and iso- to hyperintense on FS T2WI, with the presence of fluid-fluid levels. Inferiorly, it extends to the joint margin of the knee, while laterally, it causes cortical breach, leading to adjacent soft tissue involvement and edema.

**Figure 3:**
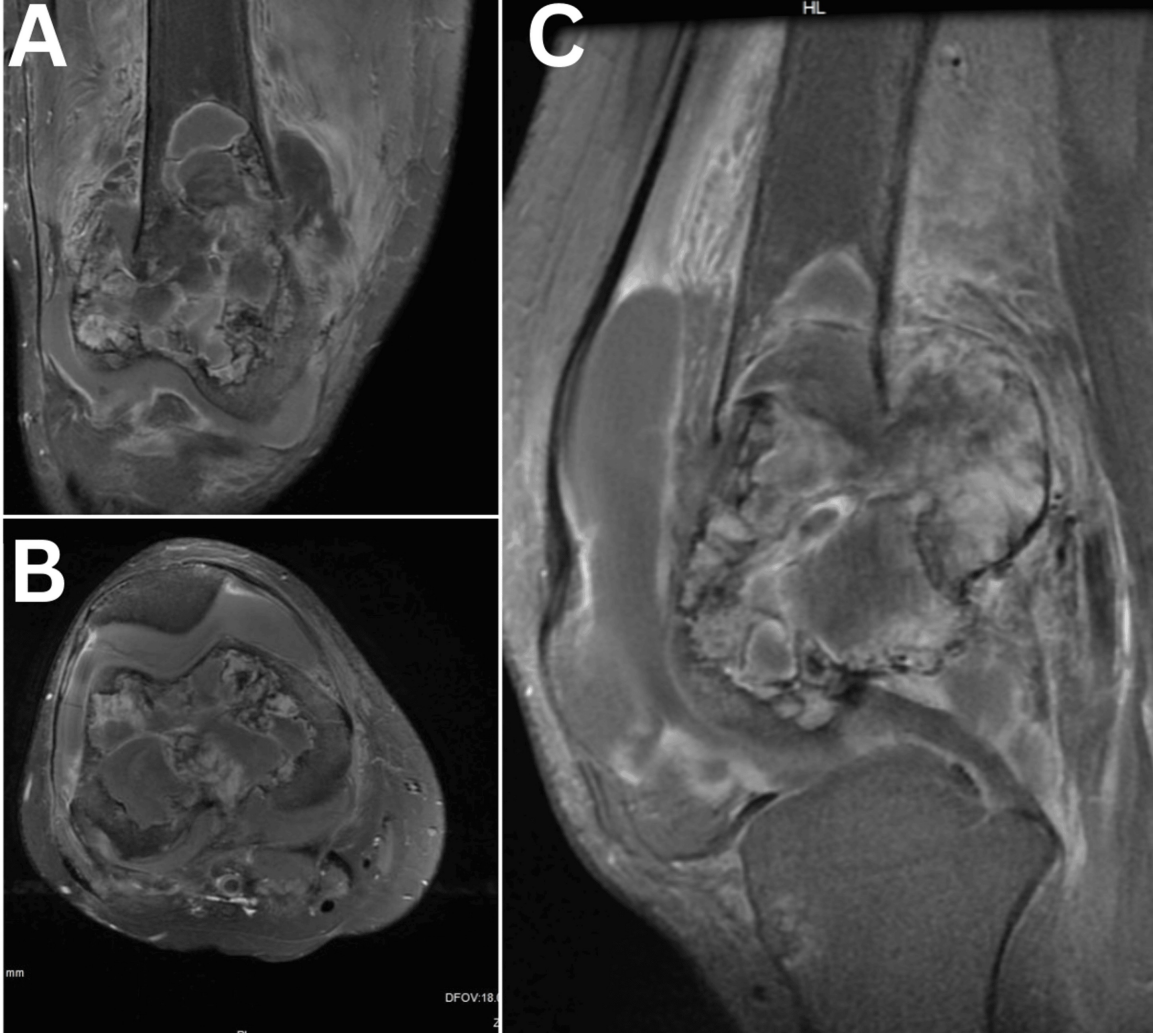
Post-contrast FS T1-weighted images in (A) the coronal, (B) axial, and (C) sagittal planes demonstrate a lesion with heterogeneous enhancement, predominantly along the periphery.

**Figure 4:**
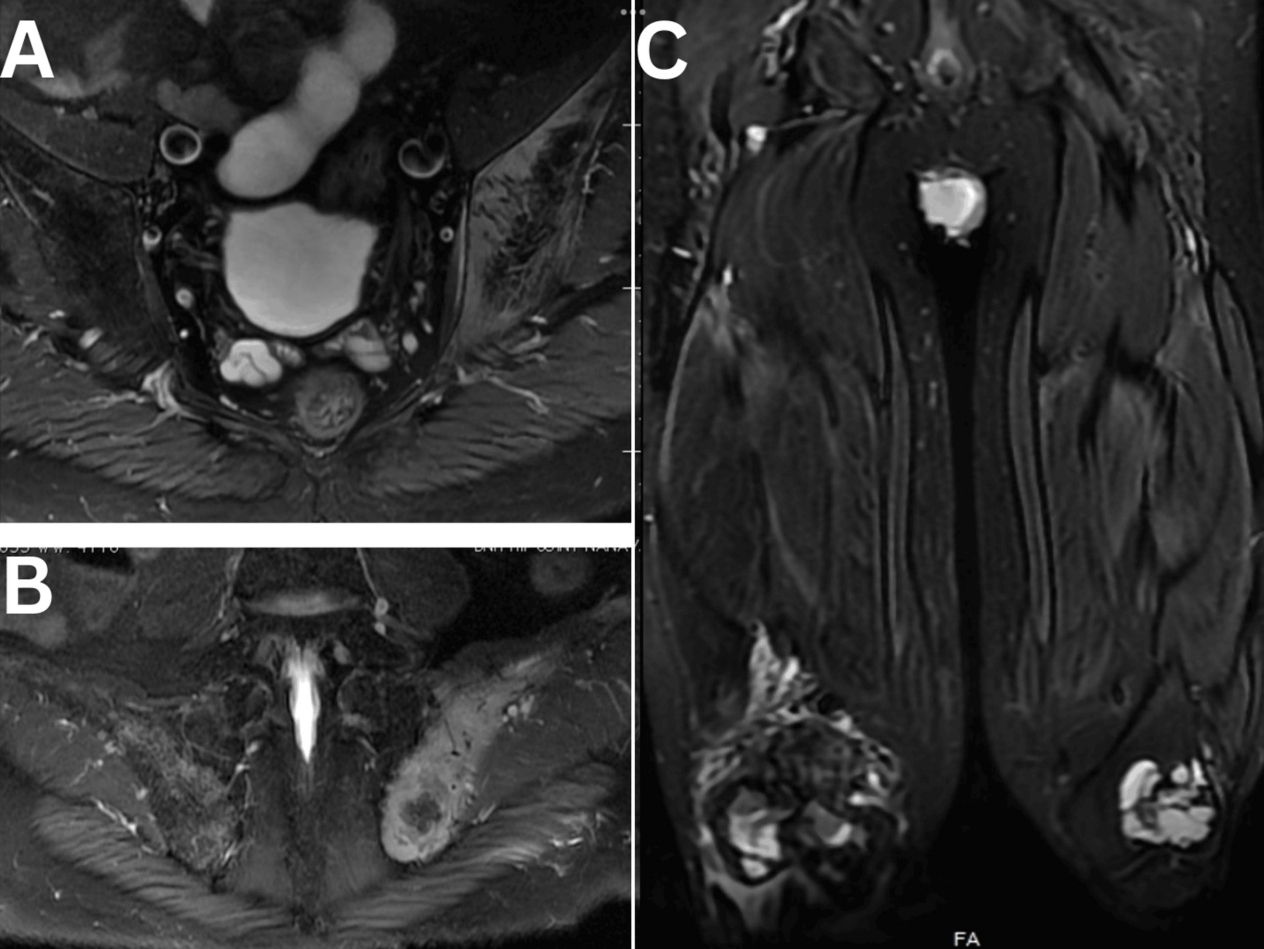
Plain MRI of the pelvis with bilateral hip joints: (A) axial fat-suppressed (FS) T2WI, (B) coronal FS T2WI. Images reveal a hyperintense lesion in the left iliac bone and sacroiliac (SI) joint, with involvement of the adjacent soft tissue and iliacus muscle. (C) Coronal FS T2WI of both thighs demonstrates multilobulated lesions in the bilateral femoral condyles with associated soft tissue involvement.

X-ray of the right femur with knee revealed lytic bone lesions with a pathological fracture (Figure 1), and MRI (Figures 2, 3) of the right knee showed heterogeneous enhancement suggestive of metastasis. MRI of the bilateral hip joint showed hyper-intense lesions in the left iliac bone with adjacent soft tissue involvement and bilateral femoral condylar involvement, further supporting the initial differential diagnosis of metastatic disease (Figure 4). Tumor markers, including cancer antigen 19-9, carcino-embryonic antigen, and prostate-specific antigen, were all within normal limits. Chest X-ray had midline trachea, clear lungs, intact equally spaced ribs, and a normal cardiac contour. However, the patient's repeat lab investigations after two days of presentation showed significantly elevated serum calcium at 14.5 mg/dL, an elevated alkaline phosphatase level of 425 U/L, and markedly elevated PTH levels at 833 pg/mL (Table 1). The high PTH level and calcium level suggest that bone resorption can be a reason for osteolysis.

**Table 1:** Laboratory findings

	Preoperative	Postoperative	Reference range
Serum calcium	14.05 mg/dl	7.15 mg/dl	8.5 to 10.5 mg/dl
Serum parathyroid hormone (PTH)	833 pg/ml	61.31 pg/ml	15 to 65 pg/ml
Serum alkaline phosphatase (ALP)	425 U/L	…….	44 to 147 U/L

**Figure 5:**
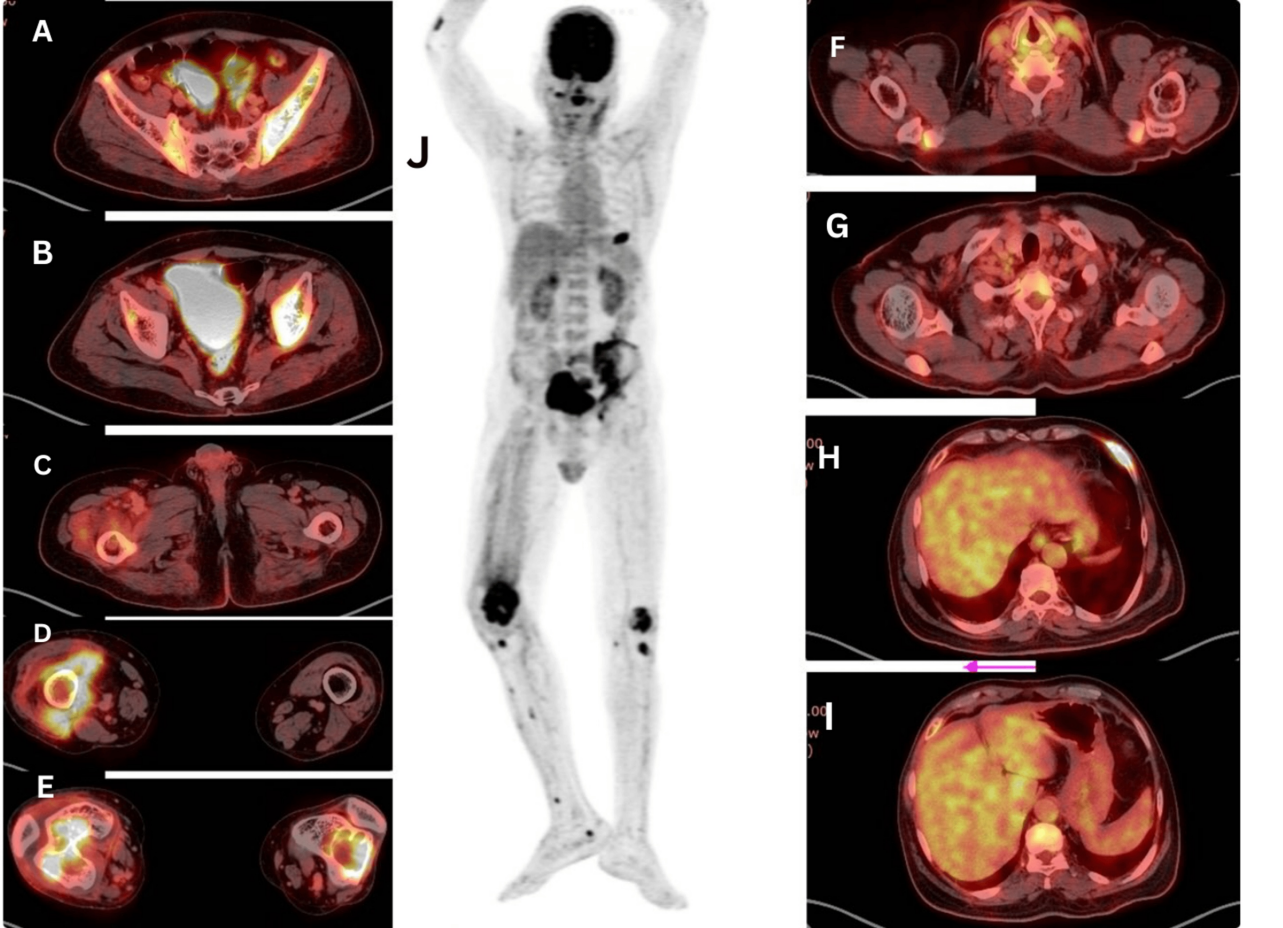
(A–C): Axial PET-CT images of the pelvis reveal a metabolically active lesion involving the left iliac bone, iliacus muscle, sacroiliac joint, and extending to the left acetabulum. (D, E): Axial images of the thigh show an extensive osteolytic lesion in the right femoral condyle extending into the articulating surface with associated soft tissue involvement, along with a similar lesion in the left femoral condyle. Axial images of the neck (F, G) and liver (H, I) regions show no metabolically active focal lesion. (J): 18F-FDG PET-CT highlights metabolically active osteolytic lesions in the left anterior sixth rib, left iliac bone, sacroiliac joint, and bilateral femoral condyles with adjacent soft tissue involvement on the right. Focal metabolic activity is also noted in the proximal bilateral tibial condyles.

**Figure 6:**
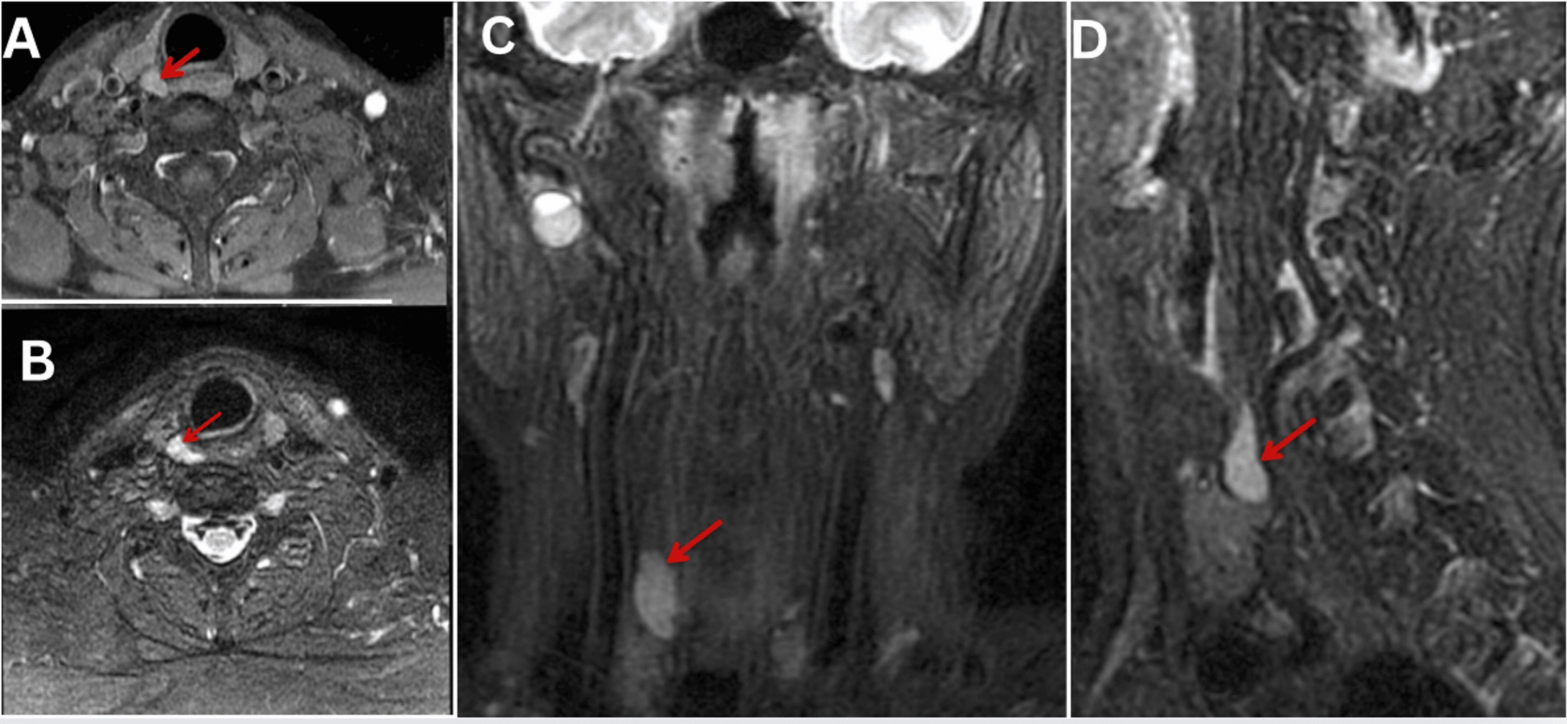
MRI neck reveal a well-defined oval isointense area in the right superior parathyroid region in axial FS T1WI (A) and hyperintense on the coronal (C) and sagittal (D) FS T2WI. Post-contrast axial FS T1WI (B) demonstrates moderate homogeneous enhancement (arrow).

**Figure 7:**
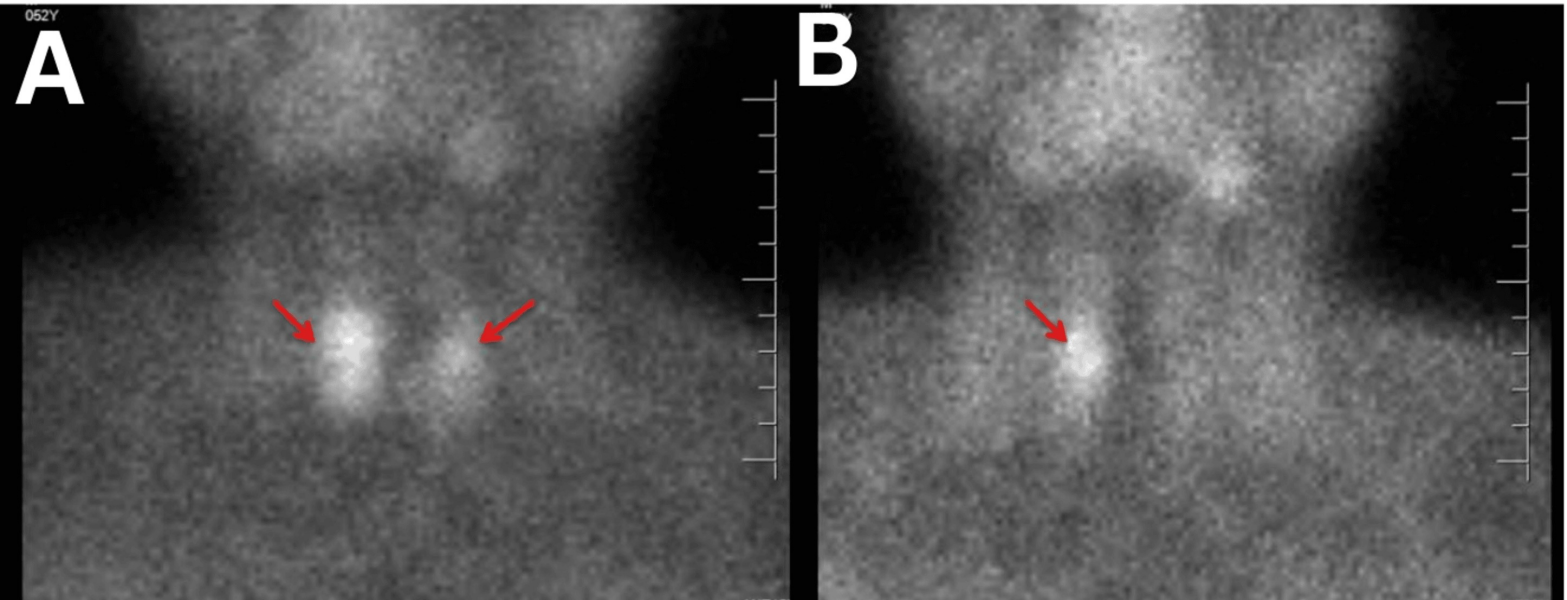
Early (A) and late (B) 99mTc-sestamibi parathyroid scintigraphy images of the neck and mediastinum. In the early phase (A), bilateral uptake is seen just below the thyroid lobes. In the delayed phase (B), only a punctate focus of persistent uptake is noted below the right thyroid lobe (arrow), corresponding to the lesion identified on MRI.

A PET scan (Figure 5) indicated metabolically active osseous lesions in both the axial and appendicular skeletons, highly suggestive of brown tumors associated with hyperparathyroidism. Further imaging with an MRI of the neck (Figure 6) identified a right parathyroid adenoma, which was confirmed by a Sestamibi scan (Figure 7).

**Figure 8:**
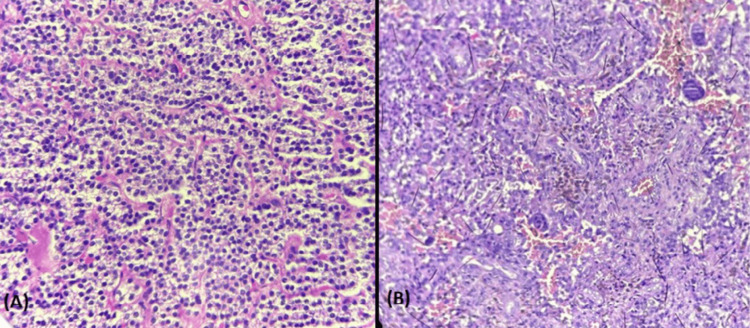
A) Parathyroid adenoma (20x, H&E): The tumor is composed of sheets of monotonous polygonal cells with round nuceli and delicate capillary network. (B) Brown tumor (20x, H&E): The tumor is composed of dense fibroblastic stroma, multinucleated osteoclastic giant cells, hemorrhage, and hemosiderin laden macrophages.

The patient was diagnosed with multiple brown tumors secondary to primary hyperparathyroidism. The surgical intervention included open reduction and internal fixation with plating and bone grafting for the pathological fracture. A biopsy of the femur revealed bone marrow infiltration characterized by reactive fibroblastic activity, multi-nucleated giant cells, areas of hemorrhage, and increased osteoblastic activity (Figure 8B). The patient subsequently underwent a right superior parathyroidectomy, and histopathological examination confirmed a right superior parathyroid chief cell adenoma (Figure 8A). In the immediate postoperative period, the PTH level was noted to drop significantly from 833 pg/ml to 61.31 pg/ml and calcium levels dropped from 14.05 to 7.15 mg/dl (Table 1). The patient suffered from severe hypocalcemia and required intravenous calcium supplementation during the initial few weeks of postoperative period, due to hungry bone disease. During the one-year follow-up visit, the patient is doing good under oral calcium and vitamin D supplements.

## Discussion

Imaging of brown tumors usually presents as multifocal osteolytic features, often raising concerns for metastatic disease. Osteolytic lesions have a wide range of differentials, not only malignant conditions like metastasis, multiple myeloma and primary bone malignancies but also benign conditions like brown tumors, giant cell tumors, and aneurysmal cysts. The location of the lesion can sometimes direct us to a diagnosis; for example, brown tumors frequently involve the jaw, pelvis, clavicle, ribs, femurs, and spine. On the other hand, the likelihood of a newly detected lesion being metastatic is 53% in the spine, 15% in the skull, and 12% in the extremities and sternum [1]. While the ribs are the most common site for new lesions, they are linked with metastasis in only 35% of cases [1]. Pathological fractures can be associated with osteolytic lesions like in this case; such fractures in weight-bearing regions are a significant risk when brown tumors compromise two-thirds of the cortex in long bones [2]. 

CT imaging in the case of brown tumors usually reveal nonspecific multi-lobulated cystic changes, often leading to misdiagnoses as metastatic carcinoma, bone cysts, or giant cell tumors. A 99mTc-MDP bone scan is helpful in differentiating brown tumors from multiple bone metastases [3]. FDG PET/CT imaging is also utilized to distinguish between malignant and benign lesions; however, a high FDG uptake can occur in benign conditions like inflammation and infections. Lesions containing osteoclast-like giant cells, such as giant cell reparative granuloma, aneurysmal bone cyst, and GCT, show increased 18F-FDG uptake, similar to benign brown tumors. The elevated uptake in brown tumors is thought to result from giant cells and the high glucose metabolism of macrophages. PET scans evaluate the metabolic activity of lesions. Parathyroid gland imaging can help in ruling out the differentials while suspecting brown tumors, but at the same time, parathyroid Sestamibi scans may also sometimes produce false negatives in detecting parathyroid conditions [4].

The histopathological work up can aid in the diagnosis, but it has some limitations. Histological differentiation between giant cell tumors (GCTs) and brown tumors of hyperparathyroidism (BTH) can be challenging, as both contain giant cells and spindle-shaped cells within a fibrous matrix. Sometimes, an important differentiating finding between brown tumors and GCTs is that the osteoclast-resembling giant cells in brown tumors are comparatively smaller in size, contain less number of nuclei, and are more evenly distributed than the giant cells in GCTs. Moreover, brown tumors are less locally aggressive than GCT, which can be correlated with some of the findings like comparatively low mitotic activity. The presence of hemosiderin-laden macrophages is the reason for the color in brown tumors [5]. The treatment approaches for these two conditions differ significantly, highlighting the importance of a clear diagnosis. This case highlights the importance of factors like age, detailed patient history, multi-system examination, and serial laboratory tests in diagnosing patients with incidental osteolytic lesions, taking into consideration all the advantages, limitations of multiple imaging modalities, and pathological workup. 

## Conclusions

This case highlights the importance of considering brown tumors in the differential diagnosis of lytic bone lesions or pathological fractures, particularly in middle-aged patients. Laboratory investigations, especially measuring serial PTH levels, are crucial for accurate diagnosis. We have discussed the various imaging modalities and the possible findings of a brown tumor, limitations of FDG-PET in differentiating brown tumor from malignancy, the role of bone scans and parathyroid scans in supporting the diagnosis, major differentials for brown tumors in histopathology, and the key differentiating findings. The diagnosis of these reversible lesions is important to avoid unnecessary interventions and guide appropriate management.
